# Three-year review of a capacity building pilot for a sustainable regional network on food, nutrition and health systems education in India

**DOI:** 10.1136/bmjnph-2020-000180

**Published:** 2021-02-01

**Authors:** Luke Buckner, Harrison Carter, Anand Ahankari, Rinku Banerjee, Somnath Bhar, Shivani Bhat, Yagnaseni Bhattacharya, Debashis Chakraborty, Pauline Douglas, Laura Fitzpatrick, Sudeshna Maitra-Nag, Sagarika Muhkerjee, Sabyasachi Ray, Ananya Roy, Aparjita Saha, Marietta Sayegh, Minha Rajput-Ray, Ianthi Tsimpli, Sumantra Ray

**Affiliations:** 1 NNEdPro Global Centre for Nutrition and Health, St John’s Innovation Centre, Cambridge, UK; 2 HALO Medical Foundation, Andur, Maharashtra, India; 3 School of Health Sciences, University of Surrey, Guildford, UK; 4 Remedy Clinic Study Group, Kolkata, India; 5 Division of Medical and Dental Education, Aberdeen Medical School, Aberdeen, UK; 6 School of Biomedical Sciences, Ulster University of Coleraine, Coleraine, Ireland; 7 Rowett Institute, University of Aberdeen, Aberdeen, UK; 8 School of Arts and Humanities, University of Cambridge, Cambridge, UK; 9 School of Humanities and Social Sciences, University of Cambridge, Cambridge, UK

**Keywords:** dietary patterns, malnutrition, nutrient deficiencies

## Abstract

**Background:**

In Kolkata (India), there are high rates of malnourished children (45.9%) under the age of three, impacting growth, organ development, function, and cognition. Mothers have a major role to play during this crucial development stage, with research showing nutrition knowledge, attitudes and practices (KAP) of mothers are important determinants of childhood malnutrition.

**Aims:**

To document 3 years of capacity building towards a sustainable nutrition education network in Kolkata, India, while assessing the ability to perform data collection in the form of needs assessments, impact assessments and capacity reviews.

**Methods:**

Descriptive review and analysis of engagement and impact from 3 years of work by the NNEdPro Global Centre for Nutrition and Health, initiating locally led nutrition education interventions. Mapping to the Indian National Nutrition Strategy was also performed to review adherence to nationwide priorities surrounding nutrition and determine the wider application potential of the network.

**Results:**

Two simultaneous projects were taken forward by a team of local healthcare professionals and student champions. Project 1—medical college workshops for medical student nutrition education with added focus on underserved populations, Project 2—preparation for a ‘Mobile Teaching Kitchen’ (MTK) in marginalised communities to empower local women as nutrition educators.

Data collection methods used for analysing markers of impact and sustainability were semi-structured interviews of the community members, and KAP questionnaires to assess response to educational sessions.

**Conclusion:**

With local support it is possible to create and sustain fieldwork for an extended period with meaningful outputs and impact. This initiative demonstrates that it is possible to use healthcare professionals, students and volunteers with low-intensity training and a low-cost approach to produce action research with considerable impact and results in rapid, reliable and robust manner.

**What this paper adds**

- An outline of methods which can be used as indicators of impact in nutrition education and research network capacity building.
- Description of 3 years of NNEdPro's establishment of a nutrition education and research network in Kolkata, India.
- A framework for learning from NNEdPro's experience in establishing a nutrition education and research network, to replicate elsewhere.

## Background

The NNEdPro Global Centre for Nutrition and Health is an inter-disciplinary think-tank, training academy and knowledge network, anchored in Cambridge, UK. It works with individuals as well as organisations interested in nutrition and health improvement via education, research, evaluation and advocacy.^1^ In February 2015, NNEdPro were invited to contribute a 2-day workshop in the 14th World Congress on Public Health (WCPH), using the opportunity to facilitate local data generation and provide insights to support local initiatives in nutrition care in Kolkata, India. This led to the creation of local projects, supervised by NNEdPro, which continue to develop.

Over the last seven decades, India underwent major economic progress demonstrated by rapid urbanisation. In 2011, approximately 31% of the Indian population were living in urban areas; almost twice as high as 1951.^2^ This shift was a result of poverty encouraging rural to urban migration. In 2009, approximately 26% of the total urban population of India lived below the poverty line,^3^ with the majority of urban poor residing in marginalised communities. Problems with overcrowding, lack of sanitation facilities, poor education and income avenues, and malnutrition coupled with poverty, are known to give rise to various health problems which in themselves lead these marginalised groups into a vicious cycle of intergenerational poverty.^4 5^

Childhood malnutrition is a major health problem, within the base of these efforts (Kolkata) there are high rates of malnourished children under the age of 3 (45.9%).^6^ Optimal nutritional intake in childhood is vital to achieve healthy physical growth, organ development and function, and cognitive development.^7 8^ Mothers, being the main caregivers in this population, have a major role to play during this crucial development stage. Research has shown that nutrition knowledge, attitudes and practices (KAP) of mothers are important determinants of childhood malnutrition,^9^ warranting further attention.

During the 14th WCPH, feedback from delegates attending the NNEdPro education workshop, held in the Eastern Indian city of Kolkata, highlighted significant gaps in knowledge and data regarding the nutritional status of school-aged children and relationships with family income, maternal education, nutrition awareness, attitudes and practices of mothers specifically residing in marginalised communities. Based on previous project experiences and learnings from the conference feedback, a strategy for a project in India was developed. The aims were to enhance nutritional awareness, build understanding of nutrition research methodology and develop leadership from the pool of delegates who had expressed an interest in improving nutritional care, in ways which were sustainable and provided opportunities for individuals to support capacity building in under-resourced health systems.

The assessment at the end of this programme identified a need for research training to enable the generation of local data. The exercise also brought into focus the dependency of key healthcare workers on nutrition guidelines derived from results of studies conducted in the western world and not in the indigenous country. These confirmed data from a previously performed need assessment in 2014 in relation to nutritional care of almost 200 doctors and dietitians in Kolkata.^10^

## Aims

To document and analyse the experience of an organisation establishing a nutrition education and research network, through description of the following:

1. Capacity building exercises undertaken.
2. Data collection and review of impact indicators.
3. Mapping to the national nutritional strategy.

## Methods

This paper descriptively reviews 3 years of NNEdPro work in Kolkata, till December 2018, to establish locally led nutrition education interventions. Including a needs assessment, impact assessment and capacity review all of which have been performed with the results collated.

While initiating a network, it is important to see how this compares with national frameworks and strategies, as such one section reviews the project to the Indian National Nutrition Strategy (INNS) ‘Nourishing India’.^11^

Descriptions of each of the interventions have not been ordered chronologically, rather, they have been categorised under three key headings; developing a sustainability framework, assessing indicators of impact and capacity, and mapping outcomes to the INNS. In the first section, the two workshops that followed the 14th WCPH and subsequent creation of the NELICO champions are described. What follows is more specific information about the NELICO urban slum dwellers group that acted as the precursor for the ‘Teaching Kitchen project from which the ‘See one, Do one, Teach one’ method was created. Still on the theme of a sustainability framework, the medical colleges and impact acceleration projects are outlined to engage local future medical graduates in capacity building as well as to draw in wider stakeholders. In measuring indicators of impact and capacity there is a reflection on needs assessments and the use of knowledge, attitudes and practice questionnaires. In the results section the projects undertaken and described are mapped to the outcomes of the INNS.

## Results

**Figure 1:**
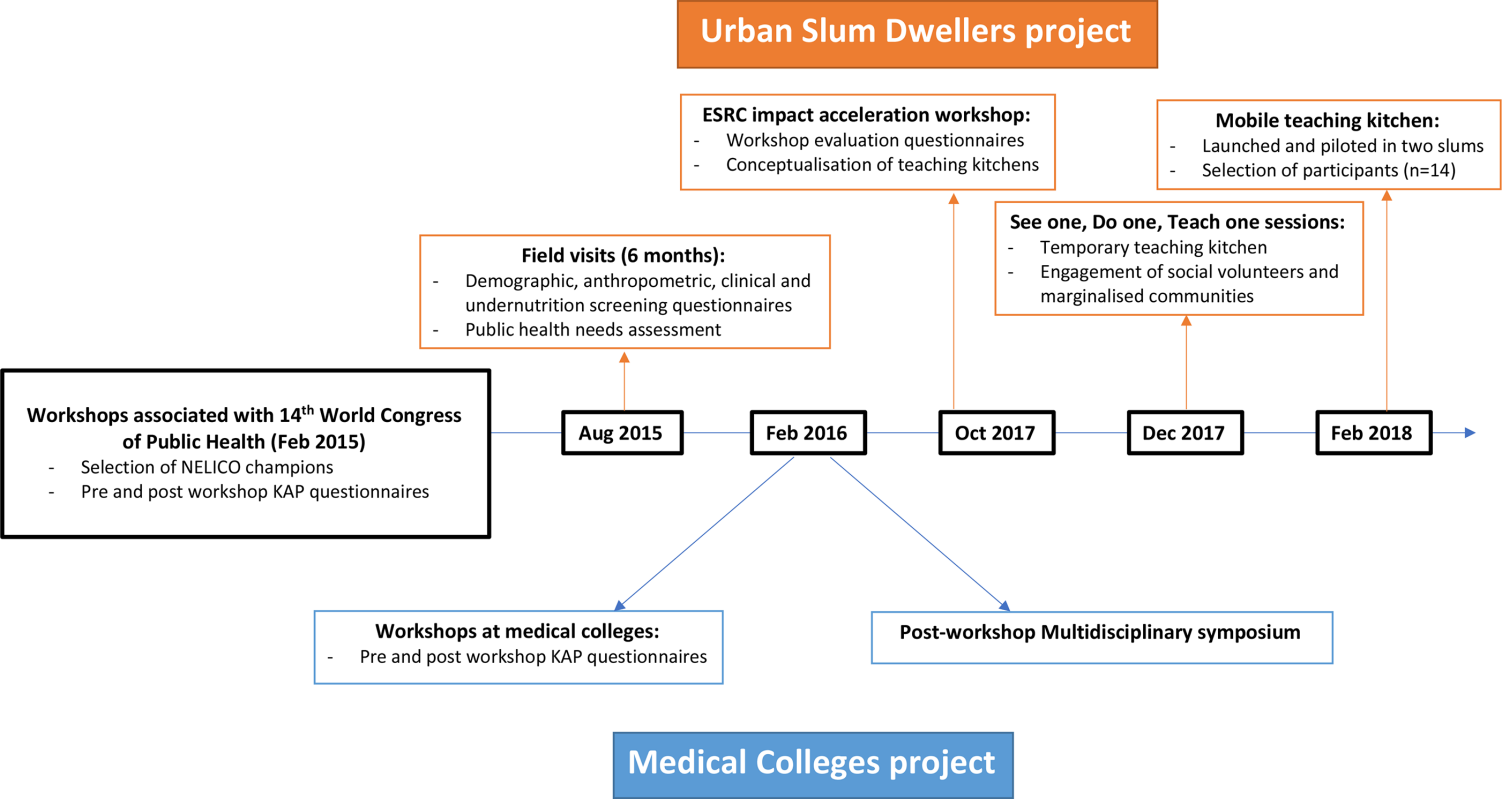
Timeline of NNEdPro’s work in India 2015–2018. ESRC, Economic and Social Research Council; KAP, knowledge, attitudes and practices.

Succeeding the first 3 years of work and data collection in India (2015–2018), our key learnings are grouped using the following sections and timeline described in figure 1.

1. Developing a sustainability framework.
2. Assessing indicators of impact and capacity.
3. Mapping outcomes to the INNS.

### Developing a sustainability framework

#### February 2015: 2-day workshop and selection of NELICO champions

**Figure 2:**
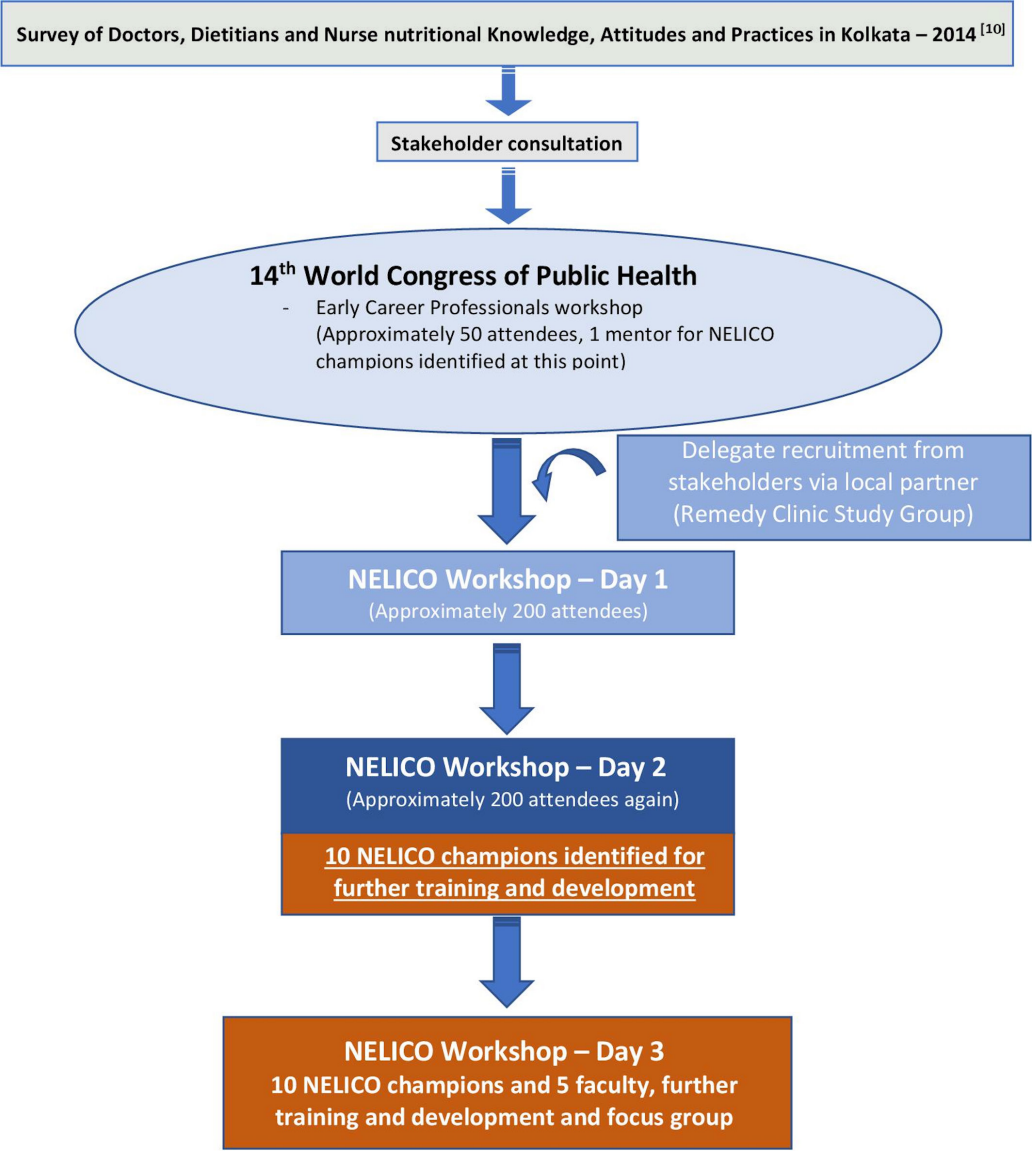
Diagrammatic outline of engagement of NELICO champions.^10^

Five members of NNEdPro were invited to Kolkata to deliver a 2-day workshop in and around the 14th WCPH in February 2015. In the build-up to this event, NNEdPro engaged in preliminary discussions with a range of organisations based in India including Halo Medical Foundation (public health field practice), Srimanta Sankara Health Sciences University (medical education and related research), All India Institute of Hygiene and Public Health (public health nutrition research), National Institute of Cholera and Enteric Diseases (laboratory research practice), Indian Institute of Management-C (healthcare leadership practices), Indian Dietetic Association (clinical nutrition and hospital practice), in which it was agreed that a diverse set of representatives, from across different nutritional relevant research and practice skillsets, would join NNEdPro for their workshop to discuss engaging in collaboration. Alongside this NNEdPro formed a working relationship with Remedy Clinic Study Group (RCSG), based in Kolkata who were key in later steps of implementing projects with local oversight. Local volunteers were also essential, and this was provided by members of the Inner Wheel Club of Greater Calcutta (figure 2).

#### Workshop day 1

On day 1 of the 2-day workshop, an initial short exercise was carried out by members of the NNEdPro Group to equip early-career public health and healthcare professionals with the knowledge, resources and skills to be ambassadors for evidence-based practice as well as introducing the principles of change management. In total, 200 healthcare professionals attended the session.

This event was followed by an interactive exercise, where the early-career professionals were divided into smaller focus groups and given the opportunity to voice their opinions/ideas and suggest strategies to tackle local nutritional problems within clinical and public health parameters.

#### Workshop day 2

On day 2, focused nutrition education training was provided with emphasis on the importance of nutrition as part of healthcare and empowering change through management and leadership techniques. A key factor of day 2 was the need for dynamic interaction between doctors, nutritionists and dietitians demonstrating that by working synergistically they can help optimise population health. This was achieved by incorporating a selection of rotational workshops consisting of five mini-rotational workshops where presentations were delivered on the importance of nutrition, nutritional care, and the importance of hydration.

During the 2-day workshop NNEdPro assessed the nutrition KAP levels of attendees before and after the nutrition education package to evaluate its effect.

#### Creation of the India NELICO champions

Towards the end of the teaching session participants were divided into multiple groups and assigned the task of designing a NELICO project to enhance nutrition education and leadership for improved clinical and/or public health outcomes relevant to local communities. ‘NELICO champions’ were then selected based on their contribution to the group work activities and their enthusiasm towards improving nutrition education. The NELICO hampions were invited to a round table event where they enriched their NELICO project ideas and decided on two projects to develop further, with the aim of raising nutritional awareness in the Indian population. They split into two teams to take forward the projects from February 2015 to February 2016, supervised by NNEdPro in partnership with RCSG and The Halo Medical Foundation.

The two teams were assisted through training workshops held in Kolkata in April 2015 and June 2015 to finalise each projects’ research methods and strengthen the proposals. Following this the NELICO champions drafted their respective key project documents including the proposal, standard operating procedure, consent and data collection forms. The proposals and supporting documents were reviewed by NNEdPro where feedback, advice and direction were provided to enhance and adapt the proposals further. Details of each projects ongoing work are described individually in the following sections, providing insight into data collected as well as the logistics of setting up and managing initiatives of this complexity. Communication was key throughout this process, particularly between events, to ensure further engagement and progress in their project. This was performed using local contacts such as RCSG, as well as email and phone call mentoring with NNEdPro.

**Figure 3:**
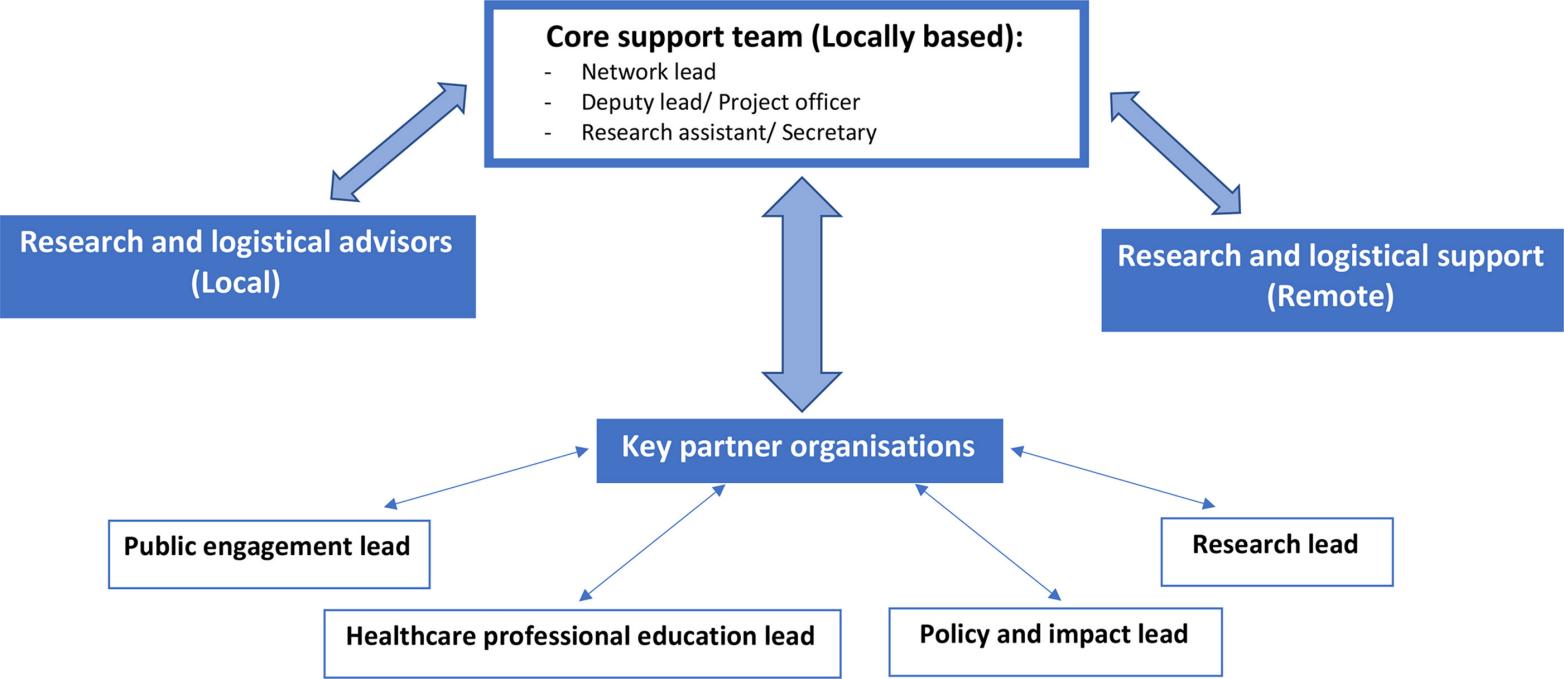
A generic format suggested for creating a regional network.

As a result of these projects, the NNEdPro India network began to grow in capacity, originally it started with five members, of which one was paid for part-time work and the rest were volunteers. By expanding to include the mentorship and inclusion of six new members from each project, this grew the capacity of the network to 17 people. This collaboration both benefited the NELICO champions in the goals of increasing nutrition awareness and increasing ability to perform research, but also the network itself benefited from mentoring them. The network grew from the funding and interest these projects brought, as well as collaboration with individuals and organisations on the peripheries. The current format is demonstrated in figure 3, where the interactions between local and remote support is demonstrated, an example of this model in action can be seen in the NNEdPro India webpage.^12^

#### Urban slum dwellers project

Early in their involvement the NELICO champions in the urban slum dwellers group identified community nutrition education as their main goal. Through scoping exercises and needs assessments they determined this was feasible. The idea of a ‘Teaching Kitchen’ was identified and developed following several adaptations to the method of education delivery. Initially a simple nutrition education session to the marginalised community was proposed, but this was restructured to involve repeated follow-up, with a method that cut through potential educational barriers. A ‘Teaching Kitchen’ was identified as a model to engage the marginalised communities in nutrition education. This involved several conversations and inputs from the remotely based NNEdPro core team, as well as visits to Kolkata to assist in planning and development of the project.

##### Launch of ‘Teaching Kitchens’ in Kolkata

In the same week of the workshop, on 24 October 2017, two NNEdPro ‘Teaching Kitchens’ were officially opened in the marginalised communities of Chetla and RG Kar Canal (Kolkata).

The team engaged deeply with the local communities and in addition to the inaugural ceremonies and cooking demonstration, they conducted Q&A sessions, and distributed a pre-prepared nutritionally balanced local meal. Its nutritional breakdown and relative amounts per recipe can be seen in online supplemental appendices 1 and 2, respectively.

10.1136/bmjnph-2020-000180.supp1 Supplementary data

10.1136/bmjnph-2020-000180.supp2 Supplementary data

#### Train-the-trainers: continuous top-up training

Members of the inner Wheel Club of Greater Calcutta (volunteers) were educated to transfer knowledge and teach appropriate methods of cooking. They were given training on affordable, local, nutritious food through cooking demonstrations and dialogue on health and hygiene from local dietitians and health professionals, beginning in 2015 and carrying through many iterative sessions till concluding sessions in July and November 2017.

#### ‘See One - Do One - Teach One’ Teaching Kitchen workshops in urban slums

From 9 December 2017 to 27 January 2018, six ‘Teaching Kitchen’ workshops were conducted using the ‘See One - Do One - Teach One’ methodology in the marginalised communities of Chetla and RG Kar Canal communities.

Dietitians, nutritionists, RCSG, NNEdPro and Inner Wheel volunteers conducted these workshops with women and children from the marginalised communities to transfer knowledge about how to create local, affordable, nutritionally balanced meals; while furthering overall engagement and awareness with the local population.

Engagement levels among children and women in both marginalised communities was promising. In addition to the impact on social and economic empowerment for women that the ‘Teaching Kitchen’ presented, the space also provided opportunities for young people and teenagers who were a highly engaged demographic. This highlighted the potential for ‘Teaching Kitchens’ as a tool for the provision of vocational education for young people and teenagers.

#### Impact acceleration workshop on nutrition

The 1-hour workshop ‘Teaching Kitchens: Next Steps in Policy and Sustainability’ was held on 25th October 2017 in Kolkata, India, organised by NNEdPro, the University of Cambridge and the RCSG, and funded by the Economic and Social Research Council (ESRC).

The workshop discussed the role of nutrition in children’s growth and development, cognitive skills and learning abilities. In addition, it advanced and implemented nutrition knowledge to improve health, well-being and society and crucially gained a consensus around the role of nutrition education and ‘Teaching Kitchens’ in empowering communities and discussed barriers or enablers of food behaviour including food availability and cultural beliefs.

#### Interactive discussion on the Teaching Kitchens project

On the 5th of February 2018, NNEdPro key collaborators from the 14th WCPH workshop visited the ‘Teaching Kitchens’ and alongside local volunteers participated in a half-day interactive discussion on the NNEdPro’s ‘Teaching Kitchen’ project. From these discussions a suggestion to add value to the programme was to refit a van to create a ‘Mobile Teaching Kitchen (MTK)’ unit, enabling the programme to access communities where lack of space presented a challenge (online supplemental appendix 3). The MTK unit was inaugurated in February 2018 in the RG Kar Canal area and in the presence of the local community that it served.

10.1136/bmjnph-2020-000180.supp3 Supplementary data

#### Medical colleges project

##### February 2016: 2-day medical college workshop—KAP

The parallel project began with approaching several medical colleges in Kolkata, inviting them to engage in a pilot intervention in autumn 2015. Of those that accepted, the deans of the medical colleges were briefed to permit the medical students to be part of the educational package.

Two sessions of education were performed on the 8th and 9th of February 2016. The bespoke nutrition intervention was based on material used for teaching medical students in the UK and adapted for the Indian context. The adapted material was taught by the NNEdPro-NELICO team which included: two nutrition dietetic graduates, one nutrition dietetic student and one medical student who worked closely with nutrition and healthcare professionals in their region and in the UK.

The 2-hour intervention focused on seven topics: nutrition for a healthy person, the importance of hydration, nutraceuticals, metabolic syndrome, diabetes management, diet in heart and disease, and nutrition support. The intervention was made interactive by having students answer questions about the material using quiz sessions at the end of each topic. Participants KAP were assessed by questionnaire before and after the intervention (online supplemental appendix 4)

10.1136/bmjnph-2020-000180.supp4 Supplementary data

##### October 2017: ESRC impact acceleration project

The project ‘The impact of an educational intervention in West Bengal, India’ was an 8-month project (15th April 2017 to 14th December 2017, later extended to 28th February 2018) sponsored by the ESRC Impact Acceleration Fund.

A key feature of the project was the co-organisation of a workshop with NNEdPro to set the foundations, as well as plan the next steps for the parallel ‘Teaching Kitchens’ project. A wide range of stakeholders in India participated, including health, academia, NGOs, civil society, local government and other key influencers in the fields of education and nutrition.

The MTK unit was further funded by the British Medical Association charities grant, Global Open Data for Agriculture and Nutrition partnership seed funding and an ESRC grant following this.

### Assessing indicators of impact and workforce capacity development

#### Needs assessments

When attempting to establish whether there would be benefit from establishing a regional network within Kolkata, it was important to first assess for any need for nutritional and public health interventions. This needs assessment focused on assessing the nutritional status of the children living in marginalised communities.

Field visits were conducted, following which nutrition workshops were organised in three marginalised communities located in three areas of Kolkata. Following clinical and anthropometric assessment, screening for malnutrition was undertaken. Semi-structured interviews with mothers were conducted to obtain data on the demographic profile of the family. This allowed a wide range of data to be obtained, while in return they were offered a health report card for participants and their children as well as referring where appropriate to local healthcare services. Furthermore, they provided nutritional advice to the community through workshops.

This method of needs assessment was able to be performed in a relatively short period of time, with low cost and provided a basic level of community data to allow repeat analysis to compare for improvements. The main reason for reduced cost was the provision of data collection and analysis by students and volunteers. For volunteers this was an opportunity to assist marginalised communities, and while the same was the case for students they also gained education and mentorship in research methods. More of a focus on the communities KAP, through questionnaires, could have been beneficial to see whether further capacity building efforts altered this from baseline; however, due to limited resources this could not be achieved.

#### Use of KAP questionnaires in measuring impact

Over the period of 2015–2018, NNEdPro assisted in developing a local group to educate and spread messages to develop interest in nutrition education for healthcare practitioners. To analyse impact of the workshops performed, KAP questionnaires were completed (table 1).

**Table 1:** Measures of impact taken between 2015 and 2018 during development of a sustainable regional network for nutrition education

Title	Date performed	Type of analysis
NELICO workshop: knowledge, attitudes and practices analysis of 2015, 2-day workshop	9th and 11th of February 2015	Preworkshop and postworkshop KAP questionnaire
Medical colleges: knowledge, attitudes and practices analysis of 2016 2-day workshop	8th and 9th of February 2016	Preworkshop and postworkshop KAP questionnaire
Landscaping research 2015–2016	2015–2016	Anthropometric, clinical assessment, demographic, questionnaire on hunger scale and cooking practices. Malnutrition screening tool

KAP, knowledge, attitude and practice.

For the two workshops this was collected with two questionnaires using the same questions, but in different orders to minimise recall bias. For the knowledge questions, the correct answers were given a score of 1 and incorrect answers were marked as 0 then added together to give a final knowledge score for each participant. For the attitude and practice questions, the responses were ranked with the best attitudes/frequent practices answers getting the highest mark and the least desirable getting the lowest.

From the results obtained it was possible to analyse the impact of an educational workshop on its participants nutrition knowledge on core topics, their attitudes around aspects of nutrition care and their self-reported practices in clinical encounters. This was performed through a relatively short intervention, before and after the educational programme and seems to hold very few negatives in its method of analysis. Data were largely collected through student or healthcare professional volunteers, with oversight from the networks core team. The overall aim of assessing both members of the marginalised communities, alongside healthcare professionals, was to assist the network in establishing the needs of the local communities and importantly the readiness of the healthcare professionals locally.

Capacity was tested, by using a combination of impact indicators such as KAP alongside engagement with the network, to assess whether a group of dedicated healthcare professionals relatively naïve to research or project leadership, could be trained to make local impacts to a publishable standard.

### Mapping outcomes to INNS

When establishing an educational network, aiming to address one of the major public health concerns worldwide, it is vital to align with indigenous strategies. In this case, working within India it was important to assess how our work addressed themes set out in the INNS as detailed in table 2.^11^

**Table 2:** Adapted from Indian National Nutrition Strategy guiding principles^11^

Principle	Summary
A life cycle approach	Recognising that there is an intergenerational cycle of undernutrition, as described in the situation analysis, a life cycle approach will be adopted, with a focus on critical periods of nutritional vulnerability and opportunity for enhancing human development potential.
Early preventative action	Recognising that growth and development deficits that compromise child health and survival and achievement of optimal learning outcomes are cumulative and largely irreversible—there will be emphasis on preventing under nutrition, as early as possible, across the life cycle.
Inclusive and gender sensitive	It will be rooted in a rights-based framework that seeks to promote the rights of women and children to survival, development, protection and participation—without discrimination. In this, strategies for ensuring social inclusion of marginalised community groups will be pursued—recognising that nutritional vulnerability is compounded by multiple deprivations—based on socioeconomic status, high burden of disease, natural factors such as floods/droughts and/or other conditions such as lack of access to services. Efforts will focus on reaching the most vulnerable and deprived.
Community empowerment and ownership	Families and communities will be enabled for improved care behaviours and nutrition of children and women, to demand quality services, to contribute to increased service utilisation and to participate in community-based monitoring.
Valuing, recognising and enhancing the contribution of anganwadi workers, helpers and ASHAs	The approach will be to improve the working conditions, skills, development pathways and motivation of Anganwadi (nutrition) workers, helpers and also Accredited Social Health Activists—a frontline team of over 3.3 million women from the local community covering 1.34 million habitations across the country—recognising that they are prime movers of social change.
Decentralisation and flexibility	Contextually relevant, decentralised approaches will be promoted, with greater flexibility at state, district and local levels for greater and sustained programme effectiveness and impact, in harmony with the approach of cooperative federalism. This will also enable utilisation of opportunities provided by the recommendations of the 14th Finance Commission with greater devolution of resources to states- mobilising and catalysing state resources and action for nutrition.
Ownership of Panchayati Raj institutions and urban local bodies	Strengthening the ownership of Panchayati Raj (village level governance) institutions and urban local bodies is a key principle—to ensure that local self-governments own, promote, monitor and sustain nutrition initiatives—effecting convergence of action at the grass roots. This is essential as the subjects allocated in the 73rd Amendment include those addressing the immediate and underlying determinants of undernutrition such as health and sanitation, family welfare, drinking water, women and child development, public distribution systems, agriculture, education, poverty alleviation and social welfare, among others. This is even more relevant in the light of the 14th Finance Commission Recommendations.
Foster innovation	Innovation will be encouraged and recognised—including through quality circles which encourage a cluster of frontline teams to identify best practices and replicate the same—with a ripple effect and widening of the innovation. Best practices will be identified and local adaptation and replication or scaling up encouraged.
Informed by science and evidence	Programme strategies will be evidence based, informed by the state of the science (as well as by the state of the practice) and updated as new evidence emerges related to nutrition, health and development.
Ensure that there is no conflict of interest	An underlying principle of action is that policy development and programme implementation must be transparent, open to public scrutiny and kept free from conflict of interest, with requisite safeguards. (This includes ensuring that representation on policy, technical advisory groups and various management committees at different levels is free from conflict of interest.)

#### Life cycle approach

The ‘urban slum dwellers’ project was aimed at educating mothers to distribute the effects to the whole family to function as change agents, and in that sense took a life cycle approach.

#### Early preventive action

Through community education of both healthcare workers through the medical college’s project as well as through lay public in the urban slum dwellers project, it is hoped that the cycle of poverty can be broken. In addition, during the health visits that conducted in parallel to the initial stages of the slum dwellers harmonisation work, doctors, dietitians and volunteers assessed the children’s anthropometry and carried out an extensive clinical examination to provide a health report card. This opportunistically identified health issues in both children and mothers which provided an opportunity for these to be addressed to offer secondary prevention benefits.

#### Inclusive and gender sensitive

In terms of project team, the initial intervention included a wide range of healthcare professionals from dietitians, laboratory scientists, doctors and students. The project aimed to target numerous parts of society including slum dwellers, empowering women and helping vulnerable children in socioeconomically deprived areas of Kolkata.

#### Community empowerment and ownership

Both NELICO interventions were encouraged to develop local leadership and ownership, which was envisioned to develop empowerment of the healthcare community locally, which then rolled into empowering lay mothers as educators in their community in the slum dwellers project. In addition, food menus designed in the MTK component of the slum dwellers project had to be locally derived and culturally sensitive. A local population of dietitians was therefore used to devise these and to support local mothers in creating their needs. The community as a whole, through training for local healthcare professionals in the NELICO project, were supported through the process of project conception and the creation of protocols and standard operating procedures. This provided a sustainable approach to learning about research methods and analysis, as well as implementation which can be carried into other areas of work or research.

#### Foster innovation

The purpose of the impact acceleration workshop was to take the solid foundations of capacity building initiatives and to complement them with innovate ideas. This led to the development of the two separate projects, which were complimentary in developing the expertise of the NELICO champions and driven by local teams. This was successful in exploring the role of nutrition in children’s growth and development, cognitive skills and learning abilities. In addition, it advanced and implemented the nutrition knowledge to improve health, well-being and society and crucially gained a consensus around the role of nutrition education and ‘Teaching Kitchens’ in empowering communities.

#### Informed by science and evidence

The approach of each intervention has developed from a base of science and evidence. As such data collection and analysis have been key to assess ongoing feasibility of both interventions. Each study employed a research methodology approach to document changes or improvement created by health or educational interventions.

#### Ensure that there is no conflict of interest

Ultimately, with funding coming from organisations globally but particularly in the UK there will be some perceived conflict of interest. However, NNEdPro works globally, with strong connections to India and are aware of the local systems and ways of working. This helped to promote the use of local skills (including human resources) to enact locally determined change. This has been facilitated with support, both financially and from a project management perspective by experts in NNEdPro.

## Discussion

Following 3 years of intervention, initiated by NNEdPro and sustained by the ongoing financial support of research grants, a local nutrition and public health educational system was established in Kolkata, India. This paper documents the path undertaken to create a sustained network that continues to thrive, by the definitions of sustainability (i) after a defined period of time, (ii) continues to be delivered, (iii) individual behaviour change is continued, (iv) adaptation continues to occur to the network while providing benefit to the individual.^13^ Comparison to the definitions set out by the WHO, suggest the network is still in its stage of growth in a life cycle, as well as being in the synthesising stage where blending of participants in order to work towards the common network goal occurs.^14^

One of the important aspects of this work by the NNEdPro in India, is that local drivers of change continue to innovate and educate through several means. The NNEdPro India network page^12^ documents a number of workshops conducted, including a *BMJ* India nutrition masterclass, which stemmed from what was initially a small group of local healthcare professionals in Kolkata. Further, through the NNEdPro India network, several discussions were conducted with vice chancellors of local medical colleges, who agreed to collaborate on increasing nutrition focus within the curriculum. Despite the initial focus on educating medical colleges, it can be seen the other arm of slum dweller education has taken the spotlight. It is largely through this that the network has used collaborations with other local partners, to bring individuals and organisations close to the project activities. From this, paid roles have been created, including a full-time project officer and intern, as well as part-time senior technical officer. The network has grown from 5 original members to 17 through initial project inclusion, to 19 core members for the ‘Teaching Kitchen’ itself, and around 51 including wider members (approximately five are renumerated or paid for their time) in the present state. Both projects continue in Kolkata, with spread to other areas within India such as Punjab, and indeed more recently the creation of the India and South-East Asia regional network to expand outside solely India. The network is increasingly independent, still using expertise from the NNEdPro core team, but managing day to day operations, development of educational articles and organisation of workshops largely from within its Kolkata based team.

From the slum dwellers team, the idea of a ‘Teaching Kitchen’ developed, and through further funding acquired it continued to develop. This educational tool continues to deliver knowledge, occupation and empowerment to a local community otherwise lacking such opportunities. Again, NNEdPro assisted and provided scientific guidance and logistical support for the MTK ‘breakout’ project, now called ‘Bhavishya Shakti’ (meaning ‘Empowering the future’). There is evidence detailing the use of hands on methods of teaching to provide nutrition education in a number of different settings, but the use in poorly educated marginalised communities has not to date been documented.^15–20^ The kitchen continues to develop, and following its initial educational intervention it was planned to visit at least monthly for continued engagement, but in fact it has grown beyond this as can be seen through activities including a microenterprise model, selling food and providing education to members of the public in Kolkata through the participants originally educated in the marginalised communities.^21^

Our review depicts the journey and development of those involved from NNEdPro in establishing the project, using the data and details collected through 3 years of work. One limitation is that this description did not extend its scope to include the opinions of those NELICO champions trained, or indeed the communities they reached for their views on sustainability or capacity of the network. While individuals still remain involved from this NELICO group, some have moved into other roles. However, their initial success has led to the momentum which drew interest from others leading to sustainable growth of the network. It remains clear from the analysis captured during the 3 years of intervention, it is difficult to quantify success. The success of this initiative is shown more clearly through the continued progress with those local professionals included, and through the research-based nutrition education they aim to continue. However, the use of questionnaires such as KAP tools, remains valuable in providing educators and researchers a way to quantify if they are having the desired impact. Overall, this demonstrates the need for forward planning of methods and techniques to analyse impact and sustainability when establishing an educational, research driven network.

It is hoped that through this paper, other researchers, healthcare professionals, or organisation will be able to reflect on our review and consider aspects of the projects outlined to replicate. This may be through adapting the proposed frameworks for an organisation or utilising data collection as markers of impact, but both benefit heavily from utilising local expertise.

## Conclusion

It is widely recognised that building and sustaining regional networks is challenging but can facilitate education and public health interventions to accelerate impact. This paper described the events leading to the creation and establishment of the NNEdPro India regional network, with exploration of indicators pertaining to early population impact and workforce capacity, mapped to a national strategy and most importantly aimed to provide a framework to replicate and learn from the experience.

This exercise demonstrates what can be achieved in generating a framework for action-research with a relatively small team, working with local collaborating organisations and individuals. Education and empowerment of local marginalised communities provide opportunities for rapid impact, while encouraging engagement and widening network membership. From here, further workforce capacity can be developed, through working with network members. This paper described how training health professionals and volunteers on the one hand to better serve marginalised communities, while also training and empowering those from underserved communities can form a two-pronged intervention for local malnutrition related inequalities. Using healthcare professionals, students and volunteers with low-intensity training needs and a low-cost approach to produce practically orientated research with potentially impactful results in rapid, reliable and robust manner. The intervention led to enhanced, research-led, nutrition education in both clinical and community settings. Furthermore, it developed local leadership and teamwork, both within and between professions, breaking down barriers between students and professionals as an example in the healthcare field. It has created a team that used the expertise and skills of different professions, including the close working of dietitians and doctors.

From the information provided, we propose a basic format that could be adapted to establish other networks with similar aims and approaches. The NNEdPro India Regional Network continues to grow, particularly with its MTK project, as well as informing modus operandi for other regional networks globally, learning from the transferrable elements of initial steps taken in Kolkata over the 2015–2017 period.

## Data Availability

Data are available upon reasonable request. Data collected throughout the three year period can be shared on reasonable request.

## References

[1] Black RE , Victora CG , Walker SP , et al . Maternal and child undernutrition and overweight in low-income and middle-income countries. Lancet 2013;382:427–51. 10.1016/S0140-6736(13)60937-X 23746772

[2] Grantham-McGregor S , Cheung YB , Cueto S , et al . Developmental potential in the first 5 years for children in developing countries. Lancet 2007;369:60–70. 10.1016/S0140-6736(07)60032-4 17208643 PMC2270351

[3] NNEdPro . NNEdPro Global Centre for Nutrition and Health [Internet], 2020. Available: https://www.nnedpro.org.uk/ [Accessed 07 Aug 2020].

[4] Registrar General and Census Commissioner . Census of India 2011 [Internet], 2011. Available: https://censusindia.gov.in/2011-prov-results/paper2/data_files/india/Rural_urban_2011.pdf [Accessed 07 Aug 2020].

[5] Ministry of Housing and Urban Poverty Alleviation of the Government of India . INDIA: URBAN POVERTY REPORT 2009 [Internet], 2009. Available: https://www.undp.org/content/dam/india/docs/india_urban_poverty_report_2009_related.pdf [Accessed 07 Aug 2020].

[6] WHO . WHO | What are the health risks related to overcrowding? WHO [Internet], 2016. Available: https://www.who.int/water_sanitation_health/emergencies/qa/emergencies_qa9/en/ [Accessed 07 Aug 2020].

[7] WHO . WHO | Sanitation and wastewater. WHO [Internet], 2019. Available: https://www.who.int/water_sanitation_health/sanitation-waste/en/ [Accessed 07 Aug 2020].

[8] Dawn A , Basu R , Fellow JR . Status and Challenges of Child Malnutrition in West Bengal with Special Reference to Area under Kolkata Municipal Corporation [Internet], 2014. Available: www.ijhssi.org [Accessed 07 Aug 2020].

[9] Fadare O , Amare M , Mavrotas G . Mother's nutrition-related knowledge and child nutrition outcomes: empirical evidence from Nigeria. PLoS One 2019;14. 10.1371/journal.pone.0212775 PMC6394922 30817794

[10] Ray S , Rajput-Ray M , Ball L , et al . Confidence and attitudes of doctors and dietitians towards nutrition care and nutrition advocacy for hospital patients in Kolkata, India. J Biomed Educ 2015;2015:1–6. 10.1155/2015/416021

[11] NITI Aayog . Nouuishing India [Internet], 2017. Available: http://niti.gov.in/writereaddata/files/document_publication/Nutrition_Strategy_Booklet.pdf [Accessed 11 May 2020].

[12] NNEdPro . India | NNEdPro [Internet, 2020. Available: https://www.nnedpro.org.uk/india [Accessed 07 Aug 2020].

[13] Moore JE , Mascarenhas A , Bain J , et al . Developing a comprehensive definition of sustainability. Implement Sci 2017;12:110. 10.1186/s13012-017-0637-1 28865479 PMC5581411

[14] World Health Organisation . Key enabling factors in effective and sustainable research networks Findings from a qualitative research study [Internet], 2016. Available: https://apps.who.int/iris/bitstream/handle/10665/205283/9789241510202_eng.pdf;jsessionid=6BD1535C45A16D1639CAAC7E92EEFBA1?sequence=1 [Accessed 28 Nov 2020].

[15] Kakareka R , Stone TA , Plsek P , et al . Fresh and Savory: integrating teaching Kitchens with shared medical appointments. J Altern Complement Med 2019;25:709–18. 10.1089/acm.2019.0091 31314556

[16] Delichatsios HK , Hauser ME , Burgess JD , et al . Shared medical appointments: a portal for nutrition and culinary education in primary Care-A pilot feasibility project. Glob Adv Health Med 2015;4:22–6. 10.7453/gahmj.2015.060 26665019 PMC4653594

[17] Herbert J , Flego A , Gibbs L , et al . Wider impacts of a 10-week community cooking skills program--Jamie's Ministry of Food, Australia. BMC Public Health 2014;14:1161. 10.1186/1471-2458-14-1161 25496263 PMC4295497

[18] Bernardo GL , Jomori MM , Fernandes AC , et al . Nutrition and Culinary in the Kitchen Program: a randomized controlled intervention to promote cooking skills and healthy eating in university students - study protocol. Nutr J 2017;16:83. 10.1186/s12937-017-0305-y 29262811 PMC5738807

[19] Eisenberg DM , Righter AC , Matthews B , et al . Feasibility pilot study of a teaching kitchen and self-care curriculum in a workplace setting. Am J Lifestyle Med 2019;13:319–30. 10.1177/1559827617709757 31105496 PMC6506983

[20] Hutchinson J , Watt JF , Strachan EK , et al . Evaluation of the effectiveness of the Ministry of food cooking programme on self-reported food consumption and confidence with cooking. Public Health Nutr 2016;19:3417–27. 10.1017/S1368980016001476 27434464 PMC10270814

[21] NNEdPro . Mobile Teaching Kitchen (MTK) [Internet], 2020. Available: https://www.nnedpro.org.uk/mtk [Accessed 16 Jun 2020].

