# Supplementary material for: Three-year review of a capacity building pilot for a sustainable regional network on food, nutrition and health systems education in India
Source: BMJ Nutr Prev Health. 2021 Feb 1;4(1):59–68. doi: 10.1136/bmjnph-2020-000180 (PMC8258077; doi:10.1136/bmjnph-2020-000180)
Supplement: Supplementary data [file bmjnph-2020-000180supp001.pdf]

## Appendix 1: Nutritional analysis of the template menu used as the educational meal

### Table 1: Trace elements and mineral composition of the 'Bhavishya Shakti' template menu

| FOOD ITEM | SODIUM (MG) | POTASSIUM (MG) | CALCIUM (MG) | IRON (MG) | ZINC (MG) | SELENIUM (MICROGRAM) | IODINE (MICROGRAM) |
|---|---|---|---|---|---|---|---|
| **INDIAN PANCAKE** | 419 | 459 | 103 | 2.9 | 2.0 | 5.0 | 16.2 |
| **MIXED VEGETABLE** | 211 | 581 | 70 | 2.6 | 0.77 | 2.3 | 1.8 |
| **SPINACH CUTLET** | 428 | 890 | 139 | 3.6 | 1.4 | 15.6 | 4.8 |
| **GREEN CHUTNEY** | 229 | 471 | 146 | 6.0 | 0.39 | 0.1 | 0.37 |
| **CHIKKI** | 22.5 | 241 | 112 | 2.0 | 1.2 | 0.45 | 3.0 |
| **TOTAL** | 1309 | 2643 | 571 | 17 | 5.7 | 23.5 | 26.1 |

Approximate weights: Indian Pancake = 165g, Mixed Vegetable = 190g, Spinach Cutlet = 165g, Green Chutney = 130g, Chikki =52g

Table 2: Vitamin composition of the 'Bhavishya Shakti' template menu

| FOOD ITEM | VITAMIN A (UG) | VITAMIN D (UG) | VITAMIN E (MG) | THIAMINE (MG) | VITAMIN B2 (MG) | VITAMIN B3 (MG) | VITAMIN B5 (MG) | VITAMIN B6 (MG) | FOLATE (MICROGRAM) | VITAMIN B12 (MICROGRAM) | VITAMIN C (MG) |
|---|---|---|---|---|---|---|---|---|---|---|---|
| INDIAN PANCAKE | 212 | 0 | 2.1 | 0.45 | 0.22 | 7.7 | 1.3 | 0.51 | 121 | 0.05 | 24.4 |
| MIXED VEGETABLE | 523 | 0 | 0.85 | 0.22 | 0.07 | 1.5 | 0.67 | 0.18 | 69 | 0 | 29 |
| SPINACH CUTLET | 266 | 0 | 4.5 | 0.17 | 0.23 | 3.1 | 0.28 | 0.26 | 179 | 0 | 54 |
| GREEN CHUTNEY | 405 | 0 | 3.8 | 0.12 | 0.24 | 1.2 | 0.29 | 0.1 | 92 | 0 | 56 |
| CHIKKI | 0.12 | 0 | 1.8 | 0.28 | 0.05 | 4.1 | 0.66 | 0.18 | 28.1 | 0 | 0 |
| TOTAL | 1406 | 0 | 13 | 1.2 | 0.81 | 17.7 | 3.2 | 1.2 | 489 | 0.05 | 163 |

Approximate weights: Indian Pancake = 165g, Mixed Vegetable = 190g, Spinach Cutlet = 165g, Green Chutney = 130g, Chikki =52g

Vitamin A = Retinol equivalent

## Table 3: Macronutrient composition of the 'Bhavishya Shakti' template menu

| FOOD ITEM | ENERGY (KCAL) | CARBOHYDRATE (G) | STARCH (G) | SUGARS (G) | PROTEIN (G) | FIBRE (G) | TOTAL FAT (G) | SATURATED FAT (G) | MUFA (G) | PUFA (G) | OMEGA-3 (G) | TRANS-FAT (G) |
|---|---|---|---|---|---|---|---|---|---|---|---|---|
| INDIAN PANCAKE | 435 | 61 | 54 | 4.5 | 19.2 | 10.9 | 13 | 2.1 | 6.9 | 2.5 | 0.17 | 0.04 |
| MIXED VEGETABLE | 123 | 12.7 | 5.9 | 6.1 | 4.5 | 5.2 | 6.1 | 0.92 | 3.6 | 1.1 | 0.05 | 0.0 |
| SPINACH CUTLET | 181 | 13.6 | 12.2 | 0.95 | 6.7 | 3.0 | 11 | 2.3 | 0.11 | 0.48 | 0.0 | 0.0 |
| GREEN CHUTNEY | 42 | 5.0 | 3.2 | 1.7 | 3.9 | 2.1 | 0.76 | 0.01 | 0.0 | 0.02 | 0.0 | 0.0 |
| CHIKKI | 253 | 26 | 2.7 | 23.3 | 6.2 | 2.2 | 13.9 | 2.6 | 6.0 | 5.0 | 0.07 | 0.0 |
| TOTAL | 1035 | 118 | 78 | 36.5 | 41 | 23.4 | 45 | 7.9 | 16.6 | 9.2 | 0.29 | 0.04 |

Approximate weights: Indian Pancake = 165g, Mixed Vegetable = 190g, Spinach Cutlet = 165g, Green Chutney = 130g, Chikki =52g

MUFA = Monounsaturated fatty acids; PUFA = Polyunsaturated fatty acids.
