# Supplementary material for: Three-year review of a capacity building pilot for a sustainable regional network on food, nutrition and health systems education in India
Source: BMJ Nutr Prev Health. 2021 Feb 1;4(1):59–68. doi: 10.1136/bmjnph-2020-000180 (PMC8258077; doi:10.1136/bmjnph-2020-000180)
Supplement: Supplementary data [file bmjnph-2020-000180supp002.pdf]

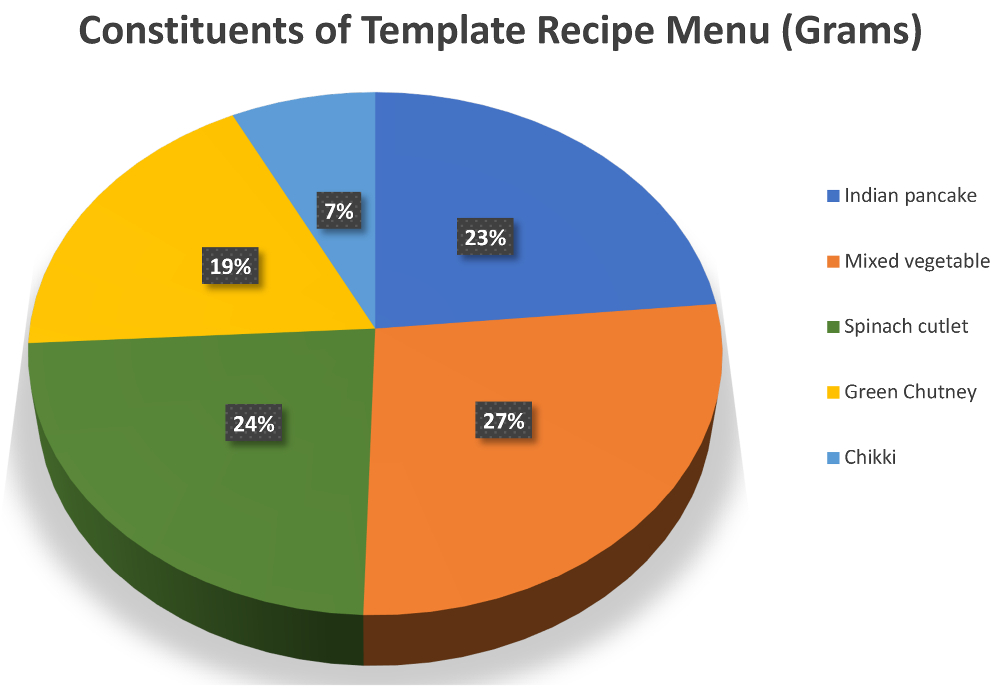

Approximate weights: Indian Pancake = 165g, Mixed Vegetable = 190g, Spinach Cutlet = 165g, Green Chutney = 130g, Chikki =52g
