# Supplementary figures and images for: Three-year review of a capacity building pilot for a sustainable regional network on food, nutrition and health systems education in India
Source: BMJ Nutr Prev Health. 2021 Feb 1;4(1):59–68. doi: 10.1136/bmjnph-2020-000180 (PMC8258077; doi:10.1136/bmjnph-2020-000180)

## Appendix 2: Diagram showing the design of the 'Mobile Teaching Kitchen' unit

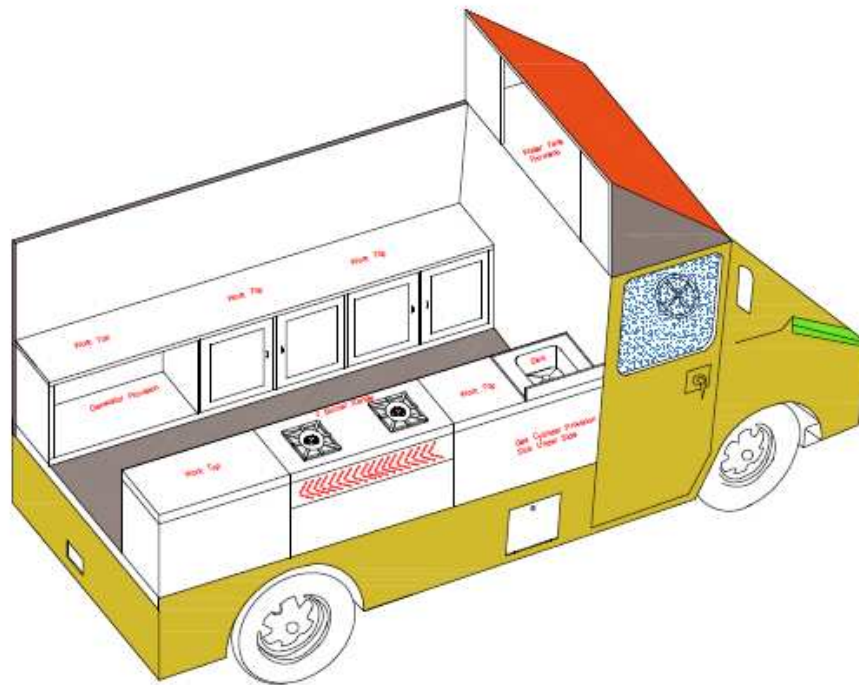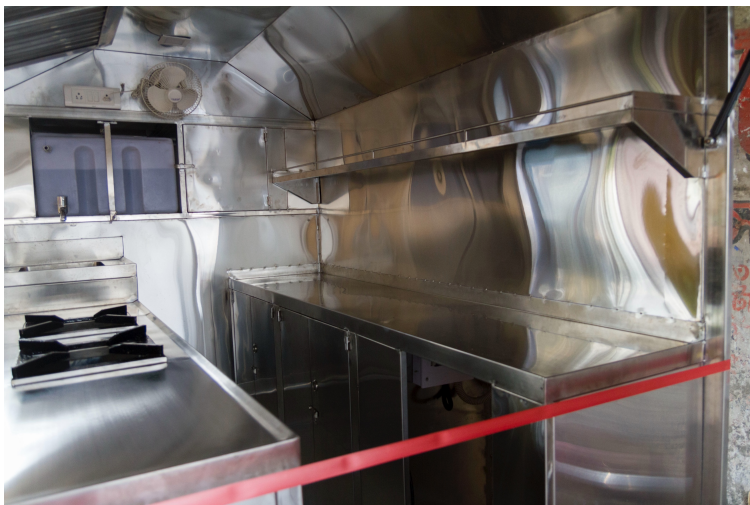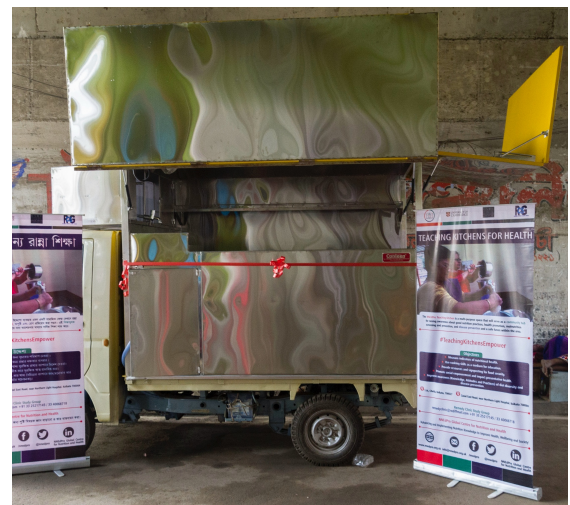

Supplement: Supplementary data [file bmjnph-2020-000180supp003.pdf]
