# Supplementary material for: Three-year review of a capacity building pilot for a sustainable regional network on food, nutrition and health systems education in India
Source: BMJ Nutr Prev Health. 2021 Feb 1;4(1):59–68. doi: 10.1136/bmjnph-2020-000180 (PMC8258077; doi:10.1136/bmjnph-2020-000180)
Supplement: Supplementary data [file bmjnph-2020-000180supp004.pdf]

Clinical Nutrition Knowledge and Attitudes Questionnaire

Please circle/ tick or highlight the correct answers

KNOWLEDGE

1. When considering nutritional support for an obese patient what would be the energy requirement?
   - a)As per patient's weight ☐
   - b)500 kcal less ☐
   - c)1000 extra kcal ☐
   - d)1000 less kcal ☐
   - ☐

2. How important is diet in management of any disease?
   - a)Not important ☐
   - b)Slightly important ☐
   - c)Very important ☐
   - d)Vital ☐

3. What is normal weight gain in pregnancy for a healthy woman of average weight?
   - a)5 kg ☐
   - b)7 kg ☐
   - c)12 kg ☐
   - d)15 kg ☐

4. Which vitamin status should be regularly monitored in patients with ileal Crohns disease?
   - a)Folic acid ☐
   - b)Thiamine ☐
   - c)Vitamin B_{12} ☐
   - d)Iron ☐

5. How much fluid should be consumed in a day?
   - a) 1-1.5L ☐
   - b) 2-2.5L ☐
   - c) 3-4L ☐
   - d) >4L ☐

6. Daily fluid requirement includes:
   - a) Only water ☐
   - b) H2O + beverages ☐
   - c) H2O + beverages + fruit juice ☐
   - d) H2O + beverages + fruit juice + soup ☐

7. What is the use of fiber in our diet?

a) Normalize bowel movement ☐
b) Lowers cholesterol level ☐
c) Helps in weight management ☐
d) All the above ☐

8. What is the calorie requirement of a normal healthy person with sedentary activity level?

a) 1000-1500 kcal ☐

b) 1500-2500 kcal ☐

c)2500-3000kcal ☐

d) over 3000 kcal ☐

9. Which one should be the ideal proportion of Carbohydrate:Protein:Fat in the diet?

a)7:2:1 ☐
b)6:3:1 ☐
c)5:3:2 ☐

d) Do not know ☐

10.Which type of Carbohydrate is beneficial for Diabetic Patients?

a) Simple ☐

b) Complex Carbohydrate ☐

c) Mixture of both ☐

d) Do not know ☐

ATTITUDE

11. A low-carbohydrate diet is the best option to lose weight

a)Strongly agree ☐

b)Agree ☐

c)Neutral ☐

d)Disagree ☐

e)Strongly disagree ☐

12. What opinion do you have regarding the fact that different disease condition require different diet?

a)Strongly agree ☐
b)Agree ☐
c)Neutral ☐
d)Disagree ☐
e)Strongly disagree ☐

13. What is your opinion regarding the fact that one should always have a balanced meal?

a)Strongly agree ☐
b)Agree ☐
c)Neutral ☐
d)Disagree ☐
e)Strongly disagree ☐

14. Do you think patients would value general nutritional advice from a Doctor?

a)Not at all ☐
b)Not much ☐
c)Somewhat ☐
d)Very much ☐

15. As one of tomorrow's doctors, would you feel equipped to give general nutritional advice to patients where appropriate or required?

a) Not at all equipped ☐
b) Inadequately equipped ☐
c) Adequately equipped ☐
d) Very well equipped ☐

16. Skipping breakfast can be a risk factor for obesity.

a) Strongly agree ☐
b) Agree ☐
c) Neutral ☐
d) Disagree ☐
e) Strongly disagree ☐

17. Stress, depression or anxiety can lead to metabolic syndrome.

a) Strongly agree ☐
b) Agree ☐
c) Neutral ☐
d) Disagree ☐
e) Strongly disagree ☐

18. Do you think supplementation is necessary other than daily diet for daily basis?

a) Strongly agree ☐
b) Agree ☐
c) Neutral ☐
d) Disagree ☐
e) Strongly Disagree ☐

PRACTICE

19. Do you skip breakfast?

a) Yes ☐
b) No ☐

20. How often do you consume fruits and vegetables in a week?

a) 1-2 days ☐
b) 3-4 days ☐
c) 5-6 days ☐
d) Every day (or 7 days) ☐

21. How often do you exercise in a week?

a) 1-3 days ☐
b) 4-6 days ☐
c) Every day ☐
d) Never ☐

22. Do you have a habit of skipping meals? If No then do not attempt this question. If yes then specify which meal.

a) Breakfast ☐
b) Lunch ☐
c) Evening snack ☐
d) Dinner ☐

23. How often do you consume street food?

a) Everyday ☐
b) 3 times a week ☐
c) Once a week ☐
d) Once in 2 weeks ☐
e) Once in a month ☐
f) Never ☐

24.What amount of fluid intake you have every day ?

a) 1liter ☐
b) 2liter ☐
c) 2.5 liter ☐
d) 3 liter ☐
e) other specify ☐

Many thanks for completing this questionnaire. Are you happy for the team to contact you in future?
Yes ☐ ***No*** ☐
